# Myocardial Fibrosis in Hypertrophic Cardiomyopathy: Leveraging Quantitative Imaging and Artificial Intelligence for Precision Management

**DOI:** 10.31083/RCM49021

**Published:** 2026-07-17

**Authors:** Yaqin Yang, Yuwei Bao, Jun Zhang, Yani Liu

**Affiliations:** ^1^Department of Medical Ultrasound, Tongji Hospital, Tongji Medical College, Huazhong University of Science and Technology, 430030 Wuhan, Hubei, China

**Keywords:** hypertrophic cardiomyopathy, myocardial fibrosis, diagnostic imaging, artificial intelligence

## Abstract

Hypertrophic cardiomyopathy (HCM) is a genetic cardiac disorder characterized by asymmetric myocardial hypertrophy and is closely linked with myocardial fibrosis. The level and severity of myocardial fibrosis are independent risk factors for adverse clinical outcomes, including being closely related to sudden cardiac death, heart failure progression, and all-cause mortality. Advances in imaging technology have expanded HCM management beyond standard morphological assessment into a multidimensional paradigm that integrates molecular characterization, functional evaluation, and prognostic stratification. This paradigm shift supports an integrated clinical approach that enables early detection, targeted therapeutic intervention, and risk-adapted prevention strategies. Innovations in artificial intelligence (AI) are further transforming HCM management into an intelligent framework driven by data analytics, predictive modeling, and precision-guided interventions. Building on the pathophysiological basis of myocardial fibrosis, this review presents a comprehensive discussion of transformative developments in multimodal imaging technologies and the associated clinical applications, supported by an in-depth analysis of the recent AI-based advances in intelligent diagnostics, risk stratification, and therapies. Collectively, these concepts aim to optimize clinical decision-making and improve patient care practices.

## 1. Introduction

Hypertrophic cardiomyopathy (HCM) is a hereditary heart disease resulting in asymmetric hypertrophy of the myocardium due to variations in the protein-coding gene encoding the sarcomere. The prevalence of HCM is 0.2%–0.5% worldwide [1,2,3,4]. HCM is one of the most common causes of sudden cardiac death (SCD) in adolescents and young adults. The annual number of implanted SCD or implantable cardioverter-defibrillator (ICD) procedures is about 0.8% [5,6]. Myocardial fibrosis represents the most characteristic pathological hallmark of HCM and is closely associated with adverse clinical outcomes, including arrhythmias and heart failure [7,8]. Pathogenic mutations in *MYH7* (β-myosin heavy chain) and *MYBPC3* (myosin-binding protein C3) genes are associated with an elevated risk for early-onset HCM, particularly when occurring as compound heterozygous variants, which may exacerbate disease severity through synergistic sarcomeric dysfunction [9].

Myocardial fibrosis is an early manifestation of HCM. The early diagnosis and differential diagnosis of myocardial fibrosis are vital to the recognition and management of HCM [10]. The gold standard for diagnosing fibrosis is histopathological staining [11,12]. However, endomyocardial biopsy is not the ideal method for assessing myocardial fibrosis due to its invasive nature and high rate of false negatives. In patients with HCM, serum biomarkers of collagen fiber metabolism are correlated with disease severity and can therefore serve as markers of myocardial fibrosis in HCM patients [13]. One meta-analysis reported that patients with myocardial fibrosis had significantly elevated serum procollagen type I carboxy-terminal propeptide (PICP) levels (standard mean difference (SMD) = 0.90 (95% CI 0.40–1.40)) and procollagen type III N-terminal propeptide (PIIINP) levels (SMD = 0.83, 95% CI 0.04–1.23) [14]. Therefore, PICP and PIIINP are believed to be potential serum biomarkers for the clinical diagnosis of myocardial fibrosis.

**Fig. 1.**
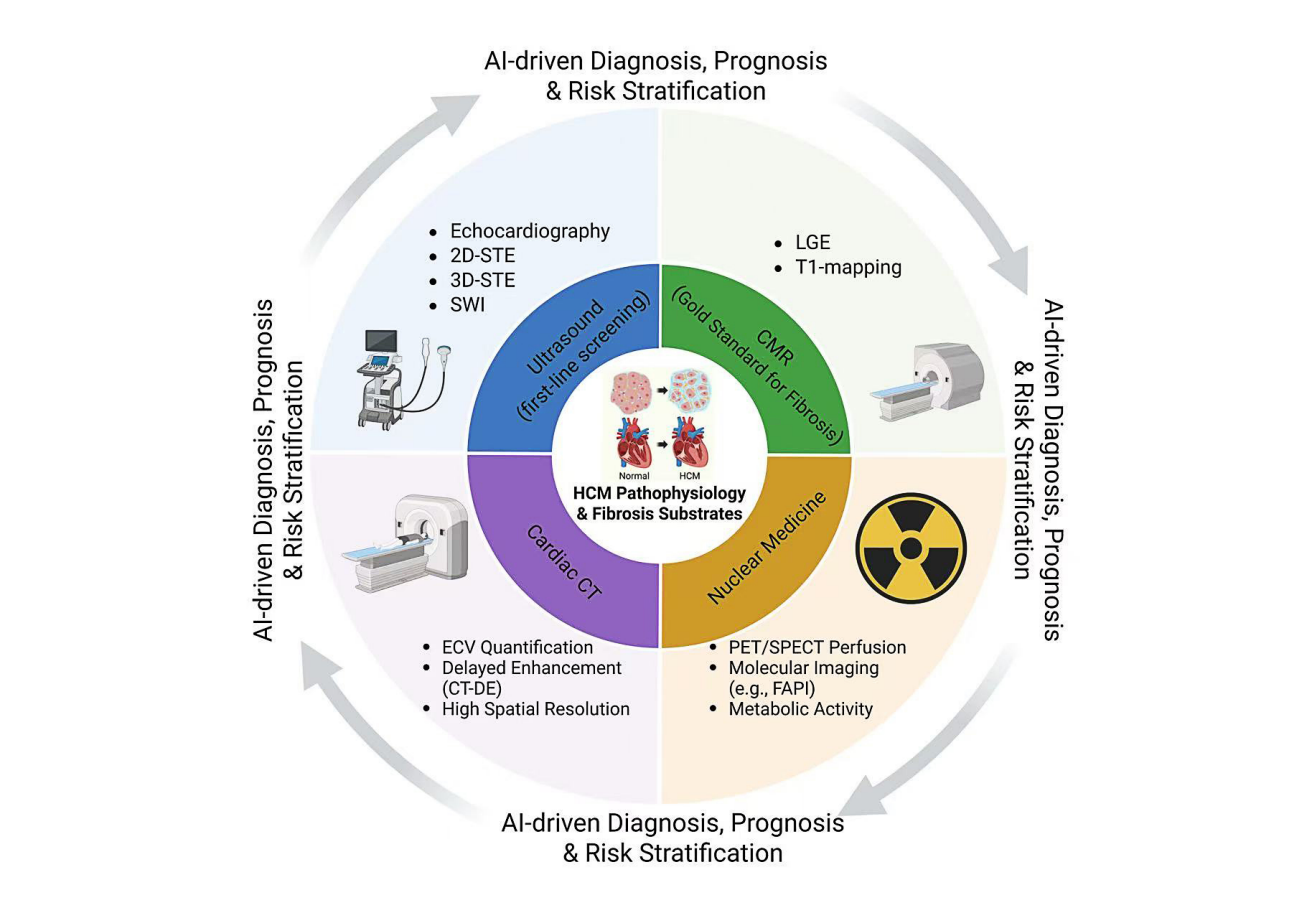
**Advances in myocardial fibrosis imaging in hypertrophic cardiomyopathy**. The figure was created in https://BioRender.com. 2D-STE, two-dimensional speckle tracking echocardiography; SWI, shear wave imaging; CMR, cardiac magnetic resonance; LGE, late gadolinium enhancement; ECV, extracellular volume; PET, positron emission tomography; SPECT, single photon emission computed tomography.

In recent years, the advent of advanced imaging modalities, such as CMR imaging, has significantly enhanced our understanding of myocardial fibrosis in HCM [15]. The latest 2024 American Heart Association/American College of Cardiology (AHA/ACC) and 2023 European Society of Cardiology (ESC) guidelines have incorporated the presence of extensive late gadolinium enhancement (LGE) into the new ICD recommendation classification algorithms to identify high-risk SCD patients [5,16,17]. High-order ultrasounds, such as strain imaging and elastography, have become useful techniques for facilitating non-invasive detection [18]. Artificial intelligence has become a revolutionary development in the diagnosis of HCM. It provides recognition of patterns of myocardial fiber disarray, automated lesion quantification, and risk stratification. When incorporated with multimodal medical imaging, it transforms conventional diagnostic paradigms by improving workflow efficiency and combining disease detection at an earlier stage. This review aims to systematically synthesize recent advancements in myocardial fibrosis research in HCM to provide a foundation for future research and improved clinical interventions (Fig. 1).

## 2. Classification and Pathophysiological Significance of Myocardial Fibrosis

### 2.1 Classification of Myocardial Fibrosis

Fibrosis is a scarring process characterized by the activation of cardiac fibroblasts, their differentiation into myofibroblasts, disruption of extracellular matrix (ECM) homeostasis, and excessive collagen deposition [19]. Histologically, fibrosis can be classified into three forms: replacement/scarring fibrosis, reactive interstitial fibrosis, and perivascular fibrosis [20]. Among these, the first two are the most common in HCM.

Reactive interstitial fibrosis is marked by increased ECM and collagen deposition between cardiomyocytes without cellular loss. This type of fibrosis is diffusely distributed throughout the myocardium and is primarily driven by chronic factors such as excessive pressure load (e.g., hypertension, obstructive HCM), inflammation, and aging [20]. It increases left ventricular (LV) stiffness and filling pressures, impairs diastolic function, and reduces exercise tolerance. The non-conductive collagen septa that develop between cardiomyocytes decelerate electrical conduction, form unidirectional blocks, and support reentrant circuits that, to a great extent, increase the risk of arrhythmias [21,22]. Perivascular fibrosis results in a deposition of collagen fibers along the coronary vessels and is characteristic of patients with hypertension. Higher perivascular collagen could result in impaired coronary vasodilator reserve and result in myocardial ischemia, which may further worsen the course of replacement fibrosis [23,24].

Replacement fibrosis occurs following cardiomyocyte death, such as after an acute ischemic injury (e.g., myocardial infarction). In HCM, patches of replacement fibrosis are often found in the interventricular septum, particularly at the insertion points of the LV and right ventricle (RV). These plaques, primarily located in the mid-myocardial layer, act as non-excitable barriers, anchoring reentrant waves and promoting arrhythmias [18].

This pathological process increases myocardial stiffness, leading to diastolic dysfunction, heart failure, and arrhythmias.

### 2.2 Pathophysiological Mechanisms of Myocardial Fibrosis

**Fig. 2.**
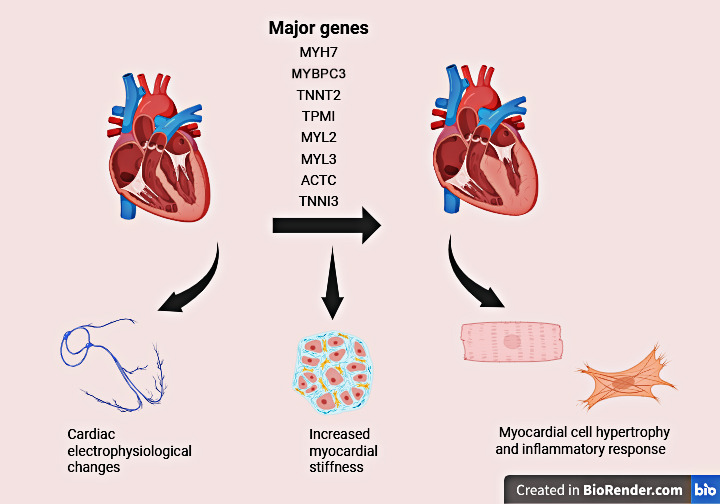
**Pathophysiological mechanisms of myocardial fibrosis in HCM**. Mutations in sarcomeric genes are the main culprits for cardiac fibrosis in hypertrophic cardiomyopathy. Cardiac electrophysiological changes, increased myocardial stiffness, myocardial cell hypertrophy, and inflammatory response participate in the formation of myocardial fibrosis in HCM. The figure was created in https://BioRender.com. HCM, hypertrophic cardiomyopathy; MYH, β-myosin heavy chain; MYBPC3, myosin-binding protein C3; TNNI3, cardiac troponin I 3; ACTC1, actin alpha cardiac muscle 1; MYL2/MYL3, myosin light chain 2/3; TPM1, tropomyosin 1; TNNT2, cardiac troponin T2.

A genetic disorder is the major cause of HCM, which results in the abnormal expression of sarcomeric protein [25,26]. These mutations lead to cardiomyocyte hypertrophy, disarray, and the activation of cardiac fibroblasts. Activated fibroblasts differentiate into myofibroblasts, disrupting ECM homeostasis and causing excessive collagen deposition, ultimately resulting in fibrosis (Fig. 2).

(1) Fibrosis significantly affects cardiac electrophysiology by disrupting the uniformity and stability of electrical conduction [27,28,29]. Replacement fibrosis forms non-conductive scar tissue, anchoring reentrant waves that trigger arrhythmias. Similarly, diffuse interstitial fibrosis creates non-conductive collagen barriers between cardiomyocytes, slowing conduction, causing unidirectional blocks, and increasing the likelihood of reentrant activity, which may lead to SCD.

(2) Fibrosis increases myocardial stiffness, reducing cardiac compliance and impairing diastolic function. This leads to increased LV end-diastolic pressure, which leads to symptoms such as pulmonary congestion [30,31,32]. Fibrosis also reduces myocardial contractility, which reduces cardiac output and may cause heart failure. Perivascular fibrosis increases myocardial injury by disrupting coronary vasodilator reserve, leading to ischemia and increased structural damage [15].

(3) Fibrosis interacts with other pathological processes, including cardiomyocyte hypertrophy and inflammatory responses. Hypertrophy increases cardiac workload, promoting the progression of fibrosis [33,34]. Inflammatory responses further activate fibroblasts, accelerating the synthesis and deposition of collagen [35]. In turn, fibrosis exacerbates cardiomyocyte injury and dysfunction, creating a vicious cycle of pathological progression [36,37].

## 3. Imaging Techniques to Assess the Progress of Myocardial Fibrosis in Hypertrophic Cardiomyopathy

### 3.1 Cardiac Magnetic Resonance (CMR)

#### 3.1.1 What Is Late Gadolinium Enhancement Cardiac Magnetic Resonance Imaging (LGE-CMR)

**Fig. 3.**
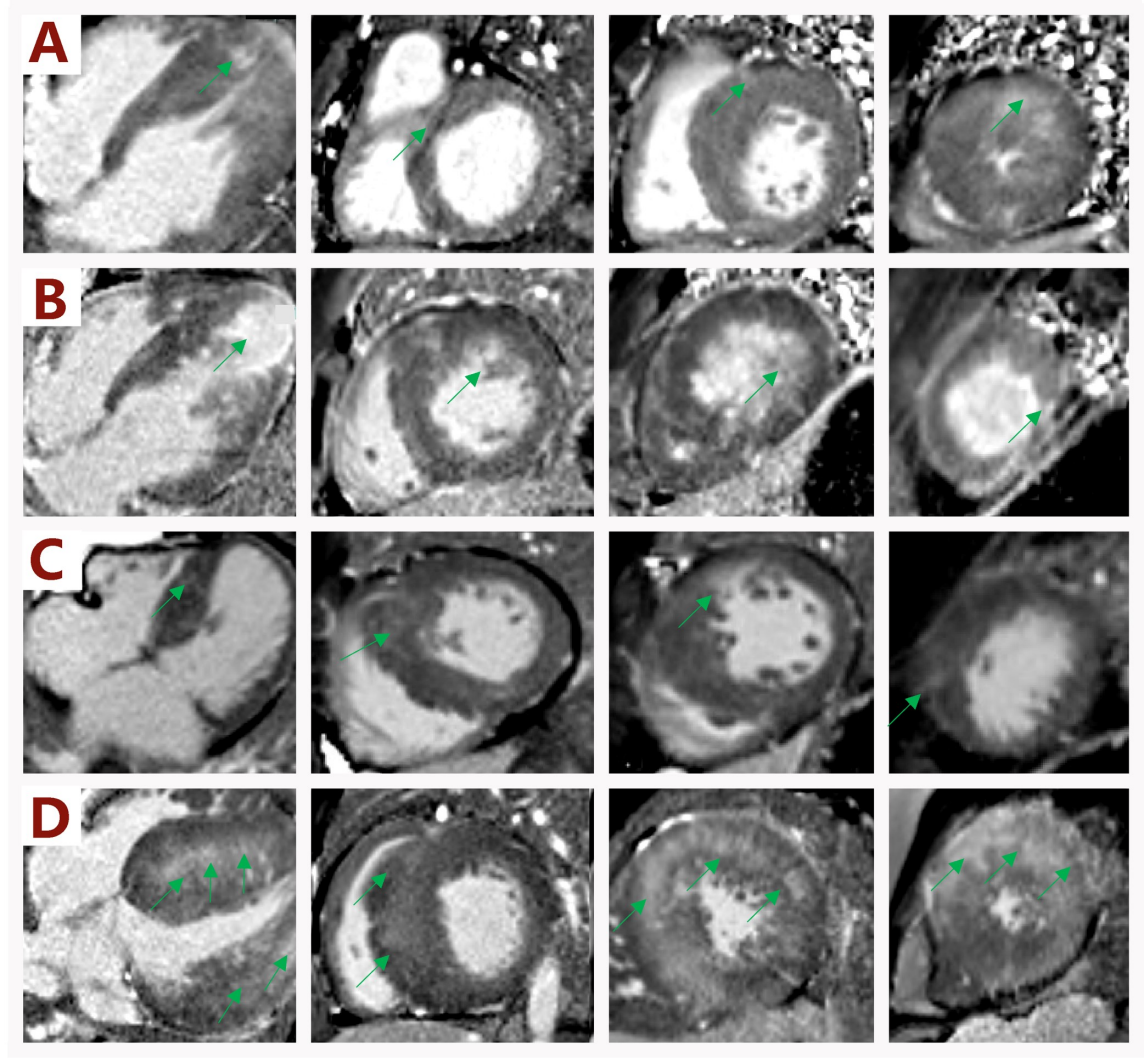
**CMR delayed enhancement imaging (LGE-CMR) has demonstrated four typical types of myocardial fibrosis distribution in patients with hypertrophic cardiomyopathy**. (A) Apical hypertrophic cardiomyopathy: LGE shows diffuse cloud-like and patchy delayed enhancement from the base to the apical myocardium, with enhancement predominating in the apical portion, suggesting widespread fibrosis. (B) Apical hypertrophic cardiomyopathy with ventricular aneurysm: LGE enhancement is mainly distributed in the mid-segment septum and right ventricular insertion with obvious enhancement of the sub-endocardium of the apical segment and the myocardium of the apical segment, and a ventricular aneurysm is present at the apical segment. (C) Interventricular septal hypertrophic cardiomyopathy: LGE shows multiple patchy delayed enhancements of the myocardium from the basal segment to the middle of the interventricular septum, which is focally striated. (D) Left ventricular diffuse hypertrophic cardiomyopathy: LGE shows multiple segments of the left ventricular myocardium with patchy abnormal enhancement signal shadow. A small area of low signal shadow is seen in the area of enhancement in the interventricular septum, suggesting widespread myocardial fibrosis with local inhomogeneous changes. Green arrows indicate the regions of myocardial fibrosis in different types of hypertrophic cardiomyopathy. Source: images from our hospital’s cardiovascular surgery department from 2022 to 2024. LGE-CMR, late gadolinium enhancement cardiac magnetic resonance imaging.

CMR provides extensive information about cardiac morphology, ventricular function, and myocardial tissue characteristics. LGE is initially found with replacement fibrosis after a myocardial infarction. With the recognition of myocardial fibrosis possibly providing a key arrhythmogenic substrate in HCM, the application of LGE-CMR for visualizing and quantifying myocardial fibrosis in HCM patients has sparked interest among clinicians following the development of techniques with increased MRI resolution [38]. LGE-CMR is a technique used to detect myocardial scarring caused by replacement or interstitial fibrosis [39] (Fig. 3). It is a robust, independent predictor of adverse outcomes such as SCD and progression to heart failure symptoms [40,41,42]. Gadolinium-based contrast agents, which accumulate excessively in myocardial damage induced expanded extracellular spaces, produce enhanced signals on CMR [43]. LGE is reported to be associated with the increased risk of malignant arrhythmias, sustained ventricular tachycardia, and ventricular fibrillation [44,45,46,47].

#### 3.1.2 Location of the Myocardial Fibrosis in HCM

Myocardial fibrosis has diverse distribution characteristics in hypertrophic cardiomyopathy. Left ventricular-affected HCM mainly manifests as asymmetric hypertrophy, with the hypertrophy of the interventricular septum being the main feature. The location of myocardial fibrosis in HCM helps to differentiate different types of left ventricular hypertrophy pathologies.

These types include apical HCM, mid-ventricular HCM, and some rare types of HCM. CMR has an important role in the diagnosis of apical HCM. The apical shape at the end-diastolic phase is similar to a ‘spade tip’, which is typical of apical HCM. Fibrosis of the apical ventricular wall, which is very likely to herald ventricular arrhythmias, is an indication for ICD implantation [48]. Mid-ventricular HCM is also a special type of HCM, in which the obstruction occurs within the left ventricular cavity. It is associated with visible mid-ventricular ring hypertrophy, which needs to be differentiated from myocardial infarction (aneurysm formation) due to coronary heart disease [49].

Diffuse hypertrophic HCM is a rare subtype of HCM. Because most HCM is asymmetric, symmetric hypertrophy is mostly caused by increased left ventricular afterload (such as hypertension, aortic stenosis, aortic coarctation) or metabolic diseases (such as cardiac amyloidosis). When considering the diagnosis of diffuse hypertrophic HCM, all possible secondary factors should be excluded [50].

Right ventricular-affected cardiomyopathy suggests the presence of biventricular involvement, which is associated with an increased risk of NYHA class III-IV heart failure [51]. Attention should be paid to the possibility of comorbid arrhythmogenic right ventricular cardiomyopathy (ARVC), which needs to be differentiated in combination with characteristics such as epsilon waves and right ventricular morphological abnormalities.

LGE has been found to determine the location of fibrosis and risk stratification [4]. It can improve the accuracy of the measurement of the thickness of the interventricular septum by distinguishing the muscular structure of the right ventricle (e.g., the septum of the atrium or the moderator band); detecting hypertrophy in the base of the apex and the anterior lateral free wall; and the subtle morphological features in non-LVH gene carriers [52]. In addition, LGE assists in stratifying the risk of SCD in HCM patients, helping to make decisions regarding the implantation of an ICD [53,54].

#### 3.1.3 Quantification of Myocardial Fibrosis

Segmentation of LGE-positive regions can be performed manually or via automated software (e.g., MRI analysis software) to calculate the percentage of left ventricular myocardial area occupied by LGE (LGE%) [55]. It is a direct visual analysis of the amount of localized fibrosis and a clinically established measure. In other cases, a segmental assessment technique permits quantitative analysis by dividing the myocardium into 16 or 17 segments, statistically analyzing the scale of fibrosis in specific segments, and evaluating distribution patterns of fibrosis (subendocardial, subepicardial, or transmural) according to anatomical locations [56]. In addition, quantitative evaluation of LGE image grayscale distribution, contrast, and entropy values can assess fibrosis heterogeneity and predict the risk of arrhythmias [57]. T1 mapping and extracellular volume (ECV) techniques are primarily utilized to quantify diffuse fibrosis.

#### 3.1.4 T1 Mapping and Extracellular Volume (ECV)

**Fig. 4.**
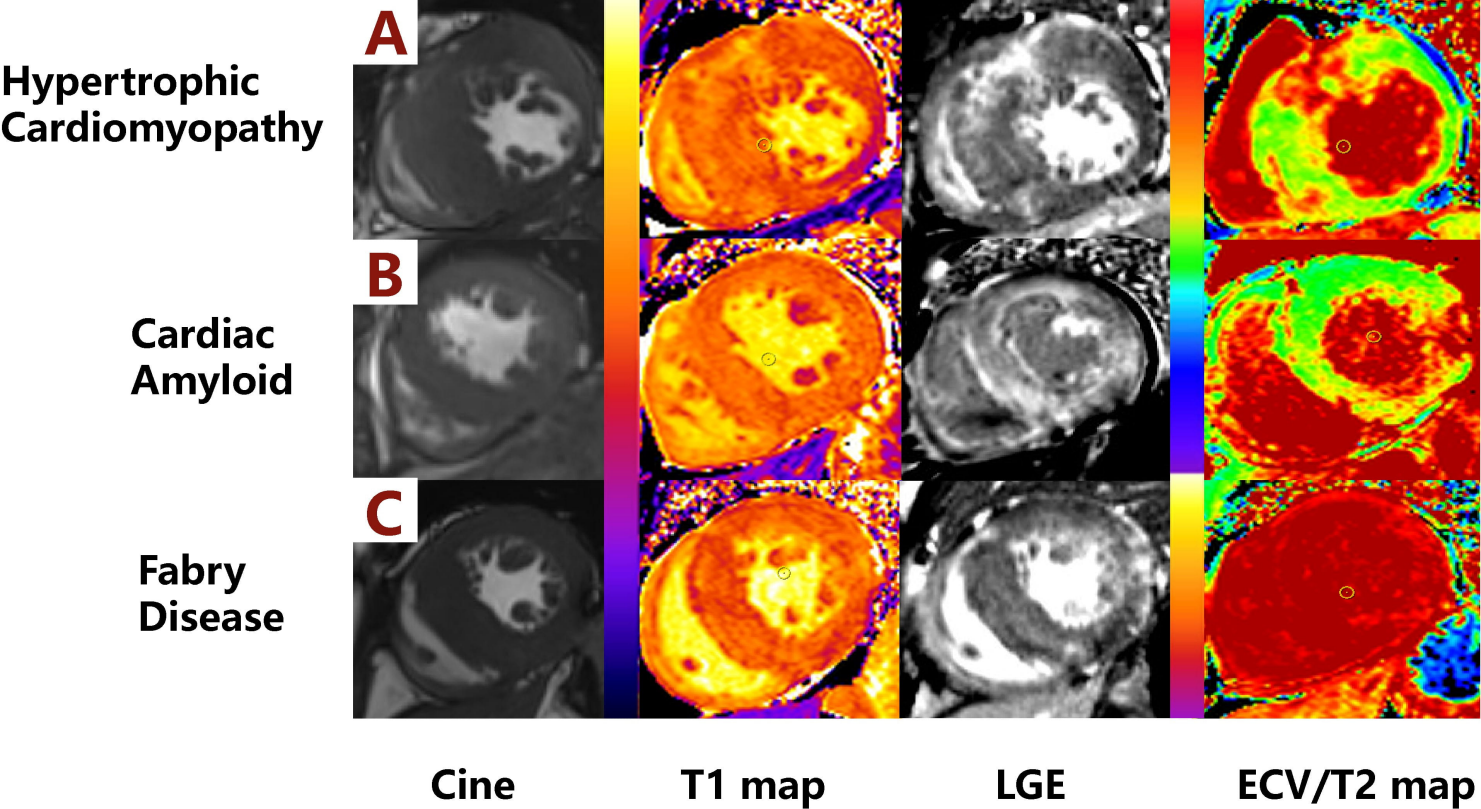
**T1 mapping and ECV in (A) hypertrophic cardiomyopathy**,** (B) cardiac amyloidosis, and (C) Fabry disease**. (A) This hypertrophic cardiomyopathy (HCM) patient demonstrated a native T1 value of 1351 ± 99 ms and an extracellular volume fraction (ECV) of 36 ± 15%, both of which were mildly elevated compared to normal ranges. Cardiac imaging revealed multiple patchy mid-myocardial late gadolinium enhancement (LGE) distributed from the basal to mid segments of the left ventricle. (B) In cardiac amyloidosis, native T1 and ECV are significantly elevated (1384 ± 61 ms and 65 ± 10%, respectively), with LGE showing diffuse delayed enhancement across multiple myocardial segments, predominantly in a subendocardial distribution and partially transmural in some regions. (C) In Fabry disease, native T1 was elevated (1384 ± 97 ms), with an ECV of 35 ± 24%; LGE was localized to the basal lateral wall of the left ventricle, showing subendocardial delayed enhancement, suggesting focal myocardial fibrosis in the advanced stage. Source: images from our hospital’s cardiovascular surgery department from 2022 to 2024.

T1 mapping is a promising new CMR technique that offers advantages over traditional LGE-CMR for more accurate quantification of diffuse fibrosis (Fig. 4) [46,58]. The T1 relaxation rate directly reflects myocardial tissue properties, which are altered in the presence of disease. Unlike LGE imaging, T1 mapping does not require comparison with normal myocardium. It can be performed on native (pre-contrast) myocardium or after the administration of contrast agents (post-contrast T1) [59]. The typical non-protein-binding gadolinium contrast agents spread throughout the extracellular space and decrease the myocardial T1 relaxation times proportional to the concentration of the agent. Using T1 mapping, fibrosis can be quantified by determining the fraction of myocardial volume occupied by the ECM (ECV fraction). Pre-contrast blood T1, calculated as synthetic ECV, is highly correlated with standard ECV and may lead to greater clinical use. T1 mapping has been demonstrated using HCM patients and has demonstrated promising results in the involvement of interstitial fibrosis in disease progression and prognosis in several studies [60,61,62,63,64].

#### 3.1.5 Limitations and Inspirations

Although it has been called the gold standard of fibrosis detection and assessment in HCM, CMR also has certain limitations. Gadolinium contrast agents have been linked to the risk of contrast-induced nephropathy, especially nephrogenic systemic fibrosis (NSF), in individuals with poor renal function (eGFR <30 mL/min/1.73 m^2^). Additionally, caution is needed for patients with cardiac implantable electronic devices (CIEDs), as pacing rate or device function may be affected during CMR acquisition [65]. Other considerations include claustrophobia, the high cost of the procedure, and the availability of CMR expertise at medical centers.

### 3.2 Cardiac Computed Tomography (CT)

Cardiac CT has gradually been applied to the assessment of myocardial fibrosis over the past decade, especially through CT delayed enhancement (CT-DE) technology to detect myocardial scarring and estimate myocardial ECV [66]. Compared to the LGE technique of cardiac magnetic resonance imaging, CT-DE has lower sensitivity but higher specificity. In clinical studies, CT-DE typically uses higher volumes of contrast agent (130 to 140 mL) and lower energy (80 kVp) to enhance contrast resolution and image quality [67]. In a study of patients with hypertrophic cardiomyopathy, the comparison between CT-DE and CMR showed a sensitivity of 79% and a specificity of 100% [68]. Despite the higher specificity of CT-DE, CMR’s LGE technique remains more widely used for clinical diagnosis.

Dual-energy CT scans at different energy levels are used to improve tissue characterization, reduce iodine load, enhance image quality, and generate virtual monoenergetic reconstructed images [69,70]. In a study, there was an increase in distinguishing different scars using low-energy (70 keV) reconstructed images compared with conventional CT-DE [71]. Consequently, the dual-energy CT technology has the potential to enhance the applications of CT-DE in measuring myocardial fibrosis.

### 3.3 Advances in Nuclear Myocardial Imaging for Myocardial Fibrosis

PET (positron emission tomography) and SPECT (single photon emission computed tomography) imaging provide information about LV fibrosis on an indirect basis, through myocardial perfusion tracers [72,73]. SPECT, however, has several limitations, such as poor spatial resolution and dependency on relative counts, and may underestimate the size of diffuse scars [73].

Contemporary PET methodology allows the measurement of absolute blood flow, and the perfusable tissue index (PTI) is an indirect measure of fibrosis. PTI is the ratio of myocardium that can affect quick water exchange, which means that at low PTI, scar burden is increased. Regardless of their potential, these methods are constrained by high equipment requirements, including on-site cyclotrons [74].

Molecular imaging, such as PET and SPECT, determines fibrogenic activity via radiolabeled tracers binding to important molecules in fibrosis [75,76]. As an example, integrin imaging is associated with fibrosis-linked cellular events and is consistent with structural and functional heart remodeling [77]. The use of radiolabeled probes in collagen or matrix metalloproteinases (MMPs) to directly visualize fibrosis is also supported by experimental work [78,79].

Although molecular imaging promises to be useful in assessing fibrosis, there are limitations, such as costly equipment, intricate tracer pharmacokinetics, and the fact that it has not been adequately validated in large-scale clinical studies [80,81]. Due to the changing technology, molecular imaging has the promise of precision medicine that can be used to diagnose diseases early, monitor diseases, and assess the efficacy of treatments in myocardial fibrosis.

### 3.4 Ultrasound Imaging in the Assessment of Fibrosis in Hypertrophic Cardiomyopathy

**Fig. 5.**
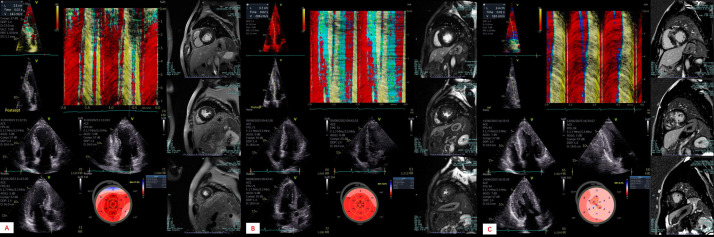
**Ultrasound imaging in HCM fibrosis assessment**. (A) Non-fibrotic HCM: LGE negative, SWV = 143 cm/s, GLS = –17.8%, normal tissue elasticity, mildly reduced systolic function. (B) Mild fibrosis HCM: LGE positive, focal distribution, SWV = 206 cm/s, GLS = –12.9%, elasticity elevated, strain decreased. (C) Severe fibrosis HCM: LGE positive, extensive distribution, SWV = 310 cm/s, GLS = –5.2%, spasticity significantly elevated, strain severely impaired. Source: images from our hospital’s cardiovascular surgery department from 2022 to 2024. HCM, hypertrophic cardiomyopathy; GLS, global longitudinal strain; LGE, late gadolinium enhancement; SWV, shear wave velocity.

Ultrasound imaging provides a non-invasive approach for assessing myocardial fibrosis in hypertrophic cardiomyopathy. Fig. 5 presents representative ultrasound imaging features of non-fibrotic, mild fibrotic, and severe fibrotic HCM, showing progressive changes in LGE status, shear wave velocity, and global longitudinal strain.

#### 3.4.1 Echocardiography

Echocardiography is a portable, inexpensive, and readily available alternative to CMR to study cardiomyopathies, especially in the diagnosis of hypertrophic cardiomyopathy. Unlike CMR, which provides direct visualization of replacement fibrosis via LGE, echocardiography relies on surrogate markers such as strain imaging to infer fibrotic burden, offering a more accessible but less specific assessment. However, echocardiography has yet to attain the ability to directly visualize fibrotic regions, particularly during the initial assessment of tissue characterization. In hypertrophic cardiomyopathy, contrast echocardiography has been utilized to detect fibrosis using the uptake of contrast agents, with locations being similar to those detected by CMR-LGE [82]. Nevertheless, the problem with hypertrophic cardiomyopathy is that the fibrosis is heterogeneous and patchy and is frequently counterembolic, small, and unevenly distributed, making it difficult to visualize and differentiate.

#### 3.4.2 Strain Imaging

Strain refers to the relative change in length of a myocardial segment compared to its baseline length, expressed as a percentage. Strain rate is the time derivative of strain and reflects the temporal characteristics of deformation [83]. Commonly used normal strains include longitudinal strain (LS), circumferential strain (CS), and radial strain (RS), which are measured along the three axes of the heart. Lengthening or thinning is represented by positive strain values, while shortening or thickening is represented by negative strain values [84]. Compared to ejection fraction (EF), global longitudinal strain (GLS) provides more information and may show abnormalities earlier in hypertrophic hearts, serving as an early indicator. The combined information from EF and GLS, along with the formed Ejection Fraction Strain Ratio (EFSR), aids in differentiating hypertrophic cardiomyopathy.

#### 3.4.3 Two-Dimensional Speckle Tracking Echocardiography (2D-STE)

Speckle tracking echocardiography (STE) helps to analyze the tissue characteristics of the disease. Multiple studies have shown that longitudinal strain (whether global longitudinal strain, GLS, or segmental longitudinal strain, SLS) is strongly correlated with the degree of myocardial fibrosis [85]. Furthermore, research has indicated that local longitudinal strain in fibrotic areas is lower than in non-fibrotic regions, making 2D-STE a valuable non-invasive tool for effectively distinguishing fibrotic and non-fibrotic lesions in HCM. Studies have also shown that SLS is significantly lower in segments with LGE compared to those without LGE, and an increased overall fibrosis burden is associated with reduced longitudinal function [86,87]. Overall, decreased longitudinal strain and increased mechanical dispersion not only reflect fibrosis but may also represent the overall pathophysiological state of HCM, such as myocardial hypertrophy, myocardial disarray, and microvascular dysfunction.

STE’s advantages include the ability to track in multiple directions, independence from angle, wide applicability, high feasibility, and fast, user-friendly (semi)automatic post-processing functions. However, the quantification in STE may not always be accurate. Despite these limitations, further standardization and pre-release testing will provide a solid foundation for the widespread application of this technology.

#### 3.4.4 Three-Dimensional Speckle Tracking Echocardiography (3D-STE)

3D-STE overcomes the inherent limitations of 2D imaging, such as apical view artifacts and errors caused by different spatial and temporal views, while also providing characteristic echocardiographic patterns to help distinguish different conditions, such as athlete’s heart from hypertrophic cardiomyopathy. However, 3D-STE has lower temporal and spatial resolution, which may affect the accuracy of measurements. This may be due to the interaction between strain and myocardial thickness in hypertrophic cardiomyopathy, as well as assumptions made by the 3D software during processing [88,89,90]. Additionally, the extent of delayed enhancement and damage in 3D global longitudinal strain was associated with the degree of myocardial hypertrophy [90]. Furthermore, three-dimensional segmental area strain (SAS) may be a potentially valuable parameter for detecting fibrosis. By optimizing spatial and temporal resolution and reducing differences between manufacturers, the potential of 3D-STE technology will be further realized.

#### 3.4.5 Shear Wave Imaging (SWI)

SWI is an ultrasound-based technique that allows for the quantitative, local, and non-invasive imaging of tissue stiffness and holds potential for assessing and diagnosing hypertrophic cardiomyopathy. Hypertrophic cardiomyopathy is also frequently associated with severe diastolic dysfunction, primarily due to myocardial fibrosis and disordered fiber arrangement. Abnormal diastolic function and changes in myocardial stiffness (MS) are seen in preserved ejection fraction (HFpEF) patients [91]. Researchers have found that SWI can accurately quantify diastolic myocardial stiffness (i.e., passive stiffness) [92,93]. Additionally, the SWI-measured anisotropy fraction (FA) helped to better understand myocardial structural changes. Villemain et al. [94] found that SWI was able to quantitatively assess MS in both healthy volunteers (HVs) and HCM-HFpEF patients. Future research may expand its application by comparing elastography parameters across different types of heart diseases to create more comprehensive diagnostic models, thereby improving the accuracy for early HCM diagnosis.

## 4. The Application of Artificial Intelligence in the Diagnosis of Hypertrophic Cardiomyopathy and Assessment of Myocardial Fibrosis

With the application of advanced imaging techniques such as cardiac magnetic resonance imaging, artificial intelligence (AI) has increasingly been used to improve early detection and the accuracy of the quantification of myocardial fibrosis. Several studies [47,95,96] have shown that AI has significant potential in the detection, prediction, and treatment of HCM-related myocardial fibrosis, and it is expected to provide more precise diagnoses and personalized treatment options in clinical practice. However, methodologically, many existing studies are limited by retrospective designs, single-center cohorts, and a lack of ethnic diversity.

### 4.1 AI in the Diagnosis of Hypertrophic Cardiomyopathy

AI technologies, specifically machine learning (ML) and convolutional neural networks (CNN), have shown enormous potential for the diagnosis of hypertrophic cardiomyopathy over the last few years [95,97,98,99]. Currently, the research of AI in HCM focuses on identifying abnormal ECG features, such as ST-T changes, abnormal Q waves, and ventricular hypertrophy, and other myocardial structural abnormalities on imaging results. Several studies [100,101,102,103] found that AI-ECG algorithms determined HCM status from the 12-lead ECG with high accuracy in diverse international cohorts. Some other studies [104,105] reported that CNN could identify HCM in patients with LVH through standard transthoracic echocardiography (TTE). Oikonomou et al. [106] developed an AI model based on a convolutional neural network that recognizes myocardial structural abnormalities by analyzing echocardiography data, which can identify 58% of HCM cases two years before the clinical diagnosis was made. The model has an AUROC of 0.90 in detecting HCM in the four-chamber view, verifying its high sensitivity and specificity. Duffy et al. [107] developed an AI algorithm that can distinguish HCM from ATTRCM, accurately identifying the characteristic left ventricular wall thickening in echocardiography, thereby reducing misdiagnosis. However, these applications face notable limitations. While Oikonomou et al. [106] reported that an AI model could identify HCM up to two years before the clinical diagnosis, the specificity of such preclinical detection remains a subject of debate. The trade-off between sensitivity and specificity must be carefully managed to avoid over-referral. The high diagnostic performance of the deep learning algorithm suggests that the use of deep learning can improve the diagnostic process in patients with LVH.

### 4.2 AI in the Assessment of Myocardial Fibrosis

Traditional methods for assessing myocardial fibrosis rely on cardiac biopsy or post-processing techniques, such as LGE imaging and T1 mapping in cardiac magnetic resonance imaging. Fahmy et al. [108] fused LGE and cine images in a CNN model, which significantly improved the robustness and accuracy of myocardial scar quantification. Compared to using only LGE, the LGE-Cine fusion model was more consistent with manual segmentation results and successfully segmented more slices (95% vs. 89%, *p* < 0.001).

However, LGE or T1 mapping is invasive, time-consuming, and requires specialized equipment. AI automatic recognition analysis in myocardial fibrosis is highly necessary to decrease the costs and procedural times. Peng et al. [109] used a machine learning algorithm (TPOT, Tree-based Pipeline Optimization Tool) to predict LGE in HCM patients, which showed great prediction ability. Nezamabadi et al. [110] developed an interpretable machine learning method based on ECG to identify and locate LV scars (areas of myocardial fibrosis) in HCM patients, which resulted in excellent prediction efficiency in validation data. These approaches offered a more convenient and precise automated tool for the evaluation of myocardial fibrosis without LGE. A machine learning model that integrates novel cardiac imaging biomarkers has been developed to exclude myocardial fibrosis in one-third of HCM patients referred for CMR using cine MRI, thus reducing unnecessary gadolinium usage [111]. Virtual native enhancement (VNE), an AI-based new technique, combines cine imaging and native T1 mapping to generate images similar to LGE without the use of contrast agents. Studies have shown that VNE has high specificity (100%) and good sensitivity (77%) for detecting scars [112].

While studies report high specificity and good sensitivity, these metrics should be interpreted with caution. Furthermore, the reliance on surrogate markers raises questions about whether these methods can truly capture the complex, patchy fibrosis characteristic of HCM. Automatic recognition using AI has wide application opportunities in myocardial fibrosis associated with HCM. Not only can it improve early detection and accurate quantification of fibrosis, but it can also offer potent prediction tools for disease progression. Nevertheless, at present, AI algorithms require verification and optimization from larger volumes of data to ensure valid use in other territories, age categories, and ethnicities, as summarized in Table 1 (Ref. [100,101,102,104,105,108,109,110,111,112]).

**Table 1.** **The application of AI in the diagnosis of HCM and assessment of MF**.

Reference	Year	Sample size	Model	Image	Main finding
Ko et al. [102]	2020	2448	CNN	ECG	ECG-based detection of HCM by an artificial intelligence algorithm can be achieved with high diagnostic performance, particularly in younger patients.
Fahmy et al. [108]	2021	191	CNN	CMR	CNN based LGE-Cine fusion can improve the robustness and accuracy of automated scar quantification.
Mancio et al. [111]	2022	1099	ML	CMR	An ML model with novel cine - based myocardial radiomic markers can rule out fibrosis in 1/3 of HCM patients referred for CMR, cutting needless gadolinium use.
Hwang et al. [105]	2022	930	CNN-LSTM	Echocardiography	The high diagnostic performance of our deep learning algorithm suggests that the use of deep learning can improve the diagnostic process in patients with LVH.
Zhang et al. [112]	2022	4271	Virtual native enhancement	CMR	VNE showed high agreement with LGE CMR in assessing myocardial scars in MI patients, in visuospatial, quantification, and with better image quality.
Peng et al. [109]	2022	135	autoML	CMR	Native T1 map analysis based on autoML correlates with LGE (+) or (–) status.
Nezamabadi et al. [110]	2023	748	ML	ECG	The model demonstrates good performance and reveals ECG features of scar in HCM.
Haimovich et al. [101]	2023	50,709	CNN	ECG	An artificial intelligence-enabled ECG model is favorable for detection and classification of LVH and outperforms clinical ECG-based rules.
Karra et al. [104]	2024	121,767	CNN	TTE	Applying artificial intelligence to the standard TTE can identify HCM patients with a reasonable accuracy.
Siontis et al. [100]	2024	4640	CNN	ECG	The AI-ECG algorithm determined HCM status from the 12-lead ECG with high accuracy in diverse international cohorts, providing evidence for external validity.

Abbreviations: AI, artificial intelligence; CMR, cardiovascular magnetic resonance; CNN, convolutional neural network; ECG, electrocardiogram; HCM, hypertrophic cardiomyopathy; LGE, late gadolinium enhancement; LVH, left ventricular hypertrophy; LSTM, long short - term memory; MF, myocardial fibrosis; MI, myocardial infarction; ML, machine learning; TTE, transthoracic echocardiogram; VNE, virtual native enhancement.

### 4.3 Clinical Translation of AI Technology

The impact that AI technology has on risk stratification and prognosis assessment is critical, and it can also be used to monitor treatment responses. These investigations are about to stimulate the revision of clinical guidelines.

Myocardial scars and fibrosis can be detected using AI algorithms based on cardiac MRI. Together with other parameters, such as the EF, they can improve the identification rate of high-risk patients [113]. The AI that estimates dynamic electrocardiograms can anticipate the danger of a severe arrhythmia (two weeks) indirectly by emphasizing the impact of fibrosis on cardiac electrical action [114].

By extracting quantitative information on fibrosis (e.g., fibrosis burden, heterogeneity) through radiomics and integrating it with clinical data (such as age and comorbidities), AI can predict the deterioration of heart function and the risk of heart failure. Studies have demonstrated that the accuracy of the AI model in predicting heart failure events can exceed 80% [113].

AI can assess the effectiveness of interventions by analyzing pre- and post-treatment imaging changes (such as the reduction in the fibrosis area) and the dynamics of biomarkers. For instance, AI can predict the likelihood of the recovery of heart function by quantifying the reduction rate of myofibroblasts. However, claims that AI will ‘reshape clinical guidelines’ must be tempered by the current lack of prospective evidence. It remains to be determined whether AI adds significant incremental prognostic value over established risk calculators.

AI technology may be incorporated into the diagnostic pathways recommended by guidelines, either replacing or supplementing traditional delayed gadolinium-enhanced MRI. Future guidelines might mandate the inclusion of AI-assisted quantitative parameters (e.g., fibrosis volume fraction) in imaging reports.

## 5. Conclusion

In recent years, the rapid development of imaging technologies and the application of AI have provided new value for the non-invasive early diagnosis, risk stratification, and observation of the effectiveness of interventions in HCM-related myocardial fibrosis. Integrating quantitative imaging phenotypes with AI-driven analytics can improve early detection, however, the sensitivity and specificity of current imaging diagnostics for HCM myocardial fibrosis still need further optimization. AI-assisted evaluation of myocardial fibrosis has the potential to reshape clinical guidelines and enhance the management of HCM patients, though its maturity should not be overstated. Future research must focus on prospective, multi-center validation.
